# A Machine Learning Method for Classification and Identification of Potato Cultivars Based on the Reaction of MOS Type Sensor-Array

**DOI:** 10.3390/s21175836

**Published:** 2021-08-30

**Authors:** Ali Khorramifar, Mansour Rasekh, Hamed Karami, Urszula Malaga-Toboła, Marek Gancarz

**Affiliations:** 1 Department of Biosystems Engineering, University of Mohaghegh Ardabili, Ardabil 56199-11367, Iran; a.khorramifar@uma.ac.ir (A.K.); hamedkarami@uma.ac.ir (H.K.); 2 Faculty of Production and Power Engineering, University of Agriculture in Kraków, Balicka 116B, 30-149 Kraków, Poland; umalagatobola@gmail.com; 3 Institute of Agrophysics, Polish Academy of Sciences, Doświadczalna 4, 20-290 Lublin, Poland

**Keywords:** potato, VOCs, olfactory machine, ANN, LDA, PCA

## Abstract

In response to one of the most important challenges of the century, i.e., the estimation of the food demands of a growing population, advanced technologies have been employed in agriculture. The potato has the main contribution to people’s diet worldwide. Therefore, its different aspects are worth studying. The large number of potato varieties, lack of awareness about its new cultivars among farmers to cultivate, time-consuming and inaccurate process of identifying different potato cultivars, and the significance of identifying potato cultivars and other agricultural products (in every food industry process) all necessitate new, fast, and accurate methods. The aim of this study was to use an electronic nose, along with chemometrics methods, including PCA, LDA, and ANN as fast, inexpensive, and non-destructive methods for detecting different potato cultivars. In the present study, nine sensors with the best response to VOCs were adopted. VOCs sensors were used at various VOCs concentrations (1 to 10,000 ppm) to detect different gases. The results showed that a PCA with two main components, PC1 and PC2, described 92% of the total samples’ dataset variance. In addition, the accuracy of the LDA and ANN methods were 100 and 96%, respectively.

## 1. Introduction

The potato is an important food crop that grows throughout the world. It is considered an essential crop in developing and developed countries, contributing to the human diet as a source of carbohydrates, proteins, and vitamins. This crop is native to South America and originates from Peru. The potato is the fourth most important food supply of human societies after wheat, rice, and maize [1]. According to the UN Food and Agriculture Organization statistics, the area under cultivation of the potato in Iran was 161 thousand hectares in 2017, while the harvested crop was about 5.1 million tons [2].

Potatoes can be consumed in various food forms, fresh or processed, including fried potatoes, mashed potatoes, potato chips, and dried granules. There are over 50 potato cultivars in the world, the most important of which are: “Agria”, with a relatively high dry matter and resistance to various pests and diseases, is used in the French Fries’ food industry. “Arinda”, with a very high-yield and resistance to internal bruising; “Almera”, with a relatively high dry matter, is suitable for fresh eating. “Burren”, with a very high-yield, is very cost-effective for cultivation and has good resistance to diseases. “Picasso”, such as the Bourne cultivar, has a high-yield and resistance to disease. “Jelly”, with a very high-yield and resistance to diseases, has a very customer-friendly yellow color and flesh. “Rumba” has high-resistance against diseases and a very high-yield. “Satina” has a very high-yield and can be cultivated in different climates. “Satne” has a high resistance to pests. “Fontane”, with a very high-yield, is suitable for processing and the French Fries’ food industry. “Sprit”, with high-yield and quality, is a customer-friendly cultivar. “Marfona” has a high-yield and is suitable for fresh eating.

The nutritional and chemical composition of potato tubers varies with cultivars, storage, growing season, soil type, pre-harvest feeding, and the analysis methods adopted [3]. In general, potatoes contain 80–70% water, 16–24% starch, and very small amounts (4%) of protein, fat, anthocyanins, minerals, and so on. Although potatoes are rich in carbohydrates, they provide significant amounts of other nutrients, such as proteins, minerals, and vitamins. Potato production is generally declining, despite an increase in market demand for fresh crops [4,5]. The production and quality of the sweetest potatoes have decreased in recent years, due to a combination of various factors, including mutations in viruses and other pathogens in potatoes [6]. Therefore, potatoes are faced with a variety of diseases. In addition, farmers have difficulty in classifying different types of plants, due to the lack of access to agricultural specialists to help to promote and educate agriculture. They cause a decrease in yield per hectare [7]. To cope with these problems, special methods have been introduced to farmers to identify and classify potato cultivars [8,9].

Traditional methods used for the determination of potato varieties were mostly based on morphological characteristics. However, the need for faster and better recognition methods was felt with the emergence of new crops [10,11,12]. Yet, the identification of different cultivars is currently accomplished by traditional and visual methods, including the observation of some characteristics of potato tubers, such as peel color, number of points in the sprouts, and, in some cases, looking at the flower color of the potato stems in the field. These methods are difficult and time-consuming tasks and are not error-free. One of the novels introduced methods in image processing techniques to identify plants based on their shape, texture, and color [13,14]. This is complemented by machine learning, which allows the machine to learn without careful planning. Since, and over the past decades, computer vision and machine learning for the identification of various diseases have been frequently used and studied. In addition, machine learning techniques can be applied to classify images. In addition, neural networks can be useful, along with image processing. The neural network is a computational framework influenced by biological neural processing. Neural networks perform useful calculations through the learning process.

Protein electrophoresis can be useful when a simple procedure, independent of high-level laboratory facilities, is required. In some cases, this method can also be applied in quality control systems. However, the requirement of special laboratory conditions (to preserve the protein, as well as the high diversity of the potato protein itself) are limitations for this method. DNA profiling: in methods that identify potato varieties through DNA, radioactivity labeling and the need for good DNA quality are among the limitations of these methods. Among these, the olfactory machine has high efficiency in classifying cultivars. It is a system with a different structure and approach, relative to other methods (image processing, neural network, etc.); it allows for the classification and identification of cultivars, is flexible, and can be applied in most agricultural products because of their odor [15,16].

Przybył et al. [17] studied two potato cultivars, Vineta and Denar, using image processing and artificial neural network techniques and concluded that they are able to identify the cultivars. This research was conducted using 4 geometric features, 7 side factors, and 29 color-determining parameters, among which, 10 factors had the highest impact on the cultivars’ identification. The optimal state of the artificial neural network used to identify these two cultivars was 18-51-2.

Azizi et al. [18] conducted an investigation on 120 potatoes of 10 different cultivars using machine vision and image processing (via the MATLAB R2012 toolbox) to identify texture and shape parameters and cultivars. At first, potato cultivars were classified using the LDA method, with an obtained accuracy of 67%. This method also failed to identify the two cultivars Agria and Savalan and misclassified the two cultivars Fontaneh and Satina, as well. They also used ANN to classify potato cultivars, in which ANN had an accuracy of 82 and 100% with one and two hidden layer(s), respectively. It was found, in this study, that different types of potatoes can be identified and classified with a very high level of accuracy using the triple properties, namely, color, textural, and morphological characteristics extracted by the machine vision using a classifying nonlinear artificial neural network. The results show the effect of artificial intelligence, including machine vision, in identifying cultivars and horticultural products that can be widely used in the food industry to achieve automation goals.

In another study, using neural networks and image processing on 5 sweet potato cultivars, researchers showed that this method is successful and could classify sweet potato cultivars with an accuracy of 100% [19].

Another study was conducted in order to grade potatoes of 5 different cultivars by their quality using a color vision machine. The researchers reported the accuracy of the LDA and MLF-NN models equal to 87 and 99%, respectively [20].

In another study, simple sequence repeats (SSR) markers were used on 34 potato cultivars grown in Canada to identify the cultivars. The results showed that the genotypes for each tested item were completely consistent, except for 4 pairs of cultivars. The accuracy of this method was obtained at 88%. The researchers also noted that, using two methods (SSR and AFLP (Amplified Fragment Length Polymorphism)), some cases show consistent results in determining potato cultivars [21].

On the other hand, for several decades, studies on the application of different types of techniques for the detection of odor have been conducted. An electronic nose is a very useful device, used to determine the difference between the smell of even similar materials [22,23,24]. For this reason, the e-nose can be regarded as a quick and simple analytical tool; it is also useful for identifying potato cultivars and will be very useful for researchers to select and produce pure cultivars and for farmers to produce uniform and certified crops. Therefore, the aim of this study is to identify potato cultivars using the olfactory machine system.

## 2. Materials and Methods

### 2.1. Sample Preparation

At first, samples of five different cultivars (Agria, Sprite, Sante, Marfona, and Jelly), were obtained from Ardabil Agricultural Research Center and kept at 4–10 °C. One day after cultivar preparation, data collection was performed. Data collection included chemical properties and cultivars’ identification using an electronic nose.

### 2.2. Extraction of Carbohydrates

The carbohydrate content of the samples was extracted using the equipment available in the central laboratory of Mohaghegh Ardabili University. It was performed using the Schlegel method. Carbohydrate was extracted using 95% ethanol, based on the sulfuric acid method from each sample. In this method, 0.200 g of the sample with 10 cc of 95% ethanol was heated in a water bath at 80 °C for 1 h. 1 cc of 0.500% phenol and 5 cc of 98% sulfuric acid were added to 1 cc of this sample. The value of absorption light by each sample from the Nanodrop spectrophotometer (Thermo Scientific™ NanoDrop™ One C, Waltham, MA, USA) with a volume of 1000 microliters was read using a cuvette. The amount of extracted carbohydrates was obtained from the standard curve by micrograms per milliliter [25].

The 100 mg/mL of glucose was prepared for the standard curve. Consecutive dilution of glucose was performed and dye development at 490 nm was controlled for different glucose concentrations. A total of 1 mL of distilled water was used as a blank. A standard curve was drawn and used to calculate the total concentration of carbohydrates.

The standard curve had a determination coefficient of 0.995 and its relationship was obtained as *y* = 0.003*x* − 0.021. Data were collected in three replications for each sample, and the amount of absorption wavelength was obtained. Then, the carbohydrates content was calculated by placement of the wavelength, in relation to the standard curve.

To obtain the carbohydrates’ content, the absorption wavelength number was placed in the relationship, *y* = 0.003*x* − 0.021 and the carbohydrates’ content was obtained by micrograms per milliliter; the values are shown in Table 1.

### 2.3. Sugar Extraction

The sugar content of each specimen was measured with three replications using a liquids’ refractometer, Model BPTR100 (Middle East System Control Co., Prisma Tech brand, Ardabil, Iran), available at Mohaghegh Ardabili University. For this, some water was extracted from each specimen, then it was poured into a micro-tube and placed in a refrigerated centrifuge (high-speed) (Model LISA France) at 1800 rpm for 2 min, following deposition of the impurities, and was separated the pure liquid (pure potato juice). It was kept, to reach ambient temperature, and then was placed on a refractometer device and its sugar content was read by Brix.

### 2.4. Electronic Nose Instrument

**Figure 1:**
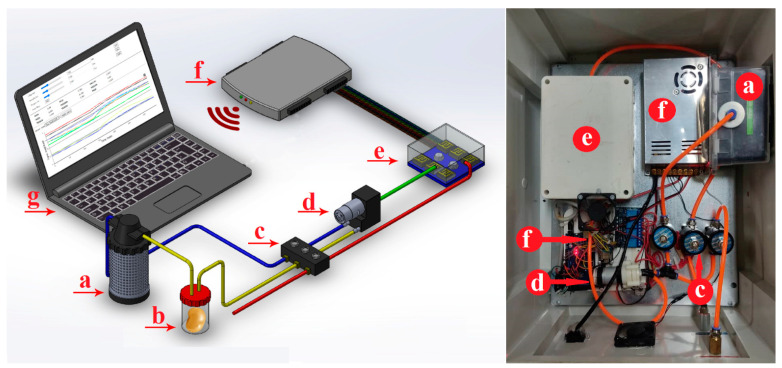
Schematic of an artificial olfactory (e-nose) system, the components of this system consist of the following parts (listed in order and direction of airflow as follows). (**a**) Air filter (activated charcoal to remove ambient-air VOC hydrocarbons), (**b**) sample headspace chamber, (**c**) solenoid air valves, (**d**) diaphragm pump, (**e**) e-nose sensor array chamber, (**f**) data acquisition recorder and wireless transmission card, and (**g**) personal computer (PC). Adapted from ref. [22].

In this research, an electronic nose made in the Department of Bio-system Engineering of Mohaghegh Ardabili University was used (Figure 1a). Additionally, 9 Metal Oxide Semiconductor (MOS) sensors with low power consumption are used in this apparatus. The sensor specifications are listed in Table 2 [22,26,27].

The first 2–4 potatoes from each cultivar were placed in the sample container (Figure 1b) for 1 day to saturate the container with the odor. Then, the sample chamber was connected to the electronic nose instrument and data collection was performed. The data were collected by the olfactory machine in such a way that first clean air was passed through the sensor chamber for 100 s to remove other odors of the sensors. The odor (gases emitted from the specimen) was then sucked out of the specimen chamber by a pump for 100 s and then directed to the sensors. Finally, to prepare the sample for further data collection, clean air was injected into the sensor chamber for 100 s [15,16,28].

According to the above steps, the output voltage of the sensors was changed, due to exposure to various gases (potato odor), and their olfactory response was collected by data collection cards. The sensor signals were recorded and stored in the computer USB gate at 1 s intervals. A fractional method was used to correct the baseline, in which noise or possible deviations were eliminated and the sensor responses were normalized and dimensionless using the following equation [16,29]:

(1) $$Y_{s}(t)=\frac{X_{s}(t)-X_{s}(0)}{X_{s}(0)}$$

where, *Y_s_*(*t*) is the normalized response, *X_s_*(0) is the baseline and *X_s_*(*t*) is the sensor response.

### 2.5. Statistical Analysis

#### 2.5.1. Analysis of Variance

The contents of sugar and carbohydrates in five different potato cultivars were obtained using a refractometer and Schlegel method, respectively.

The obtained values for sugar and carbohydrate content of five potato cultivars were analyzed using MSTATC software (Michigan State University, East Lansing, MI, USA). The statistical analyses were conducted using a completely randomized factorial test. The means were compared with Duncan’s multiple range test at 0.01 *p*-value level.

#### 2.5.2. Chemometrics and Machine Learning Modelling

**Figure 2:**
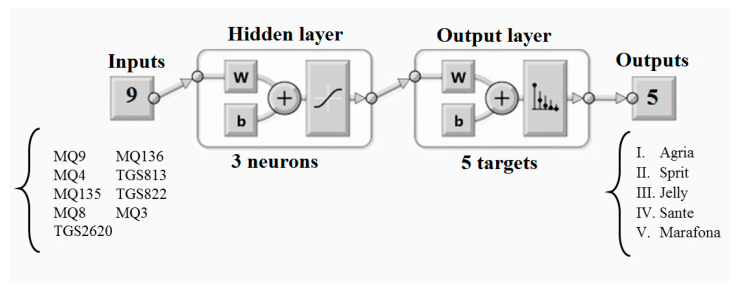
Diagrams of the two-layer feedforward models with a tan-sigmoid function in the hidden layer and a Softmax function in the output layer for electronic nose inputs. Abbreviations: W: weights; b: bias. Adapted from ref. [32].

Chemometrics uses multivariate statistics to extract useful information from complex analytical data. The chemometric used in this study began with principal component analysis (PCA) to discover the output response of the sensors and reduce the data dimension. In the next step, linear diagnostic analysis (LDA) and artificial neural network (ANN) were used to classify five potato cultivars. PCA is one of the most widely used methods to reduce statistical data. This method is an unsupervised technique used to explore and reduce the dimensions of a dataset. The analysis involves determining the variable components, which is a linear combination of many features studied [30]. A set of correlated variables becomes a new set of orthogonal variables called principal components (PCs). Each principal component is a linear combination of all primary variables. LDA is a supervised method used to find the most distinctive Eigenvectors and maximizes the ratio of the variance between and within the class and is able to classify two or more groups of samples [31]. Artificial neural network (ANN) is a computational model, based on the function and structure of biological neural networks. The information that flows through the network affects the structure of the ANN because the neural network changes or, in other words, learns, based on input and output. The common type of artificial neural network consists of three groups or layers: the first layer is connected to the hidden layer and they themselves are connected to the output layer. The activity of the input units represents the raw information that is transmitted to the network. The activity of each hidden unit is determined according to the activities of the input units and the weight on the connections between the input and the hidden units. Additionally, the behavior of the output units depends on the activity of the hidden units and the weights between the hidden units and the output. In this type of network, the hidden units are free to construct their own representations of the input. The weights between the hidden and input units are determined when each hidden unit is active; so by modifying these weights, a hidden unit can choose what it represents. One of the important applications of neural networks is pattern recognition. Pattern recognition can be implemented using a feed-forward neural network, trained in the same way. During training, the network is trained to relate the outputs to the input patterns. When a network is used, it detects the input pattern and tries to output the associated output pattern. The power of neural networks comes to life when a pattern that has no output associated with it, is given as an input. In this case, the network gives the output that corresponds to a taught input pattern that is least different from the given pattern. According to the number of sensors, nine neurons were considered for the input layer. The hidden layer will be considered with the optimal number of neurons and five output neurons will be considered according to the number of target output classes. Data were randomly selected for learning (60%), testing (20%), and validation (20%). The performance was calculated using the cross-entropy and a neuron trimming test was conducted to select the models with no under- or over-fitting, being three the most optimal number for the model (Figure 2) [32]. In addition, Unscrambler vers. 10.4 software (CAMO AS, Trondheim, Norway) was used for PCA and LDA analysis and Matlab^®^ (vers. R2014a) (Mathworks, Inc., Natick, MA, USA) was used for ANN analysis.

### 2.6. Model Evaluation Metrics

The individual performance of models 1 and 2 was assessed by the confusion matrix and the receiver performance characteristic curve (ROC). Once validated, each model was exported as an executable command in Matlab^®^ and challenged by a new testing dataset. Sensitivity, specificity, accuracy, and precision parameters were used to analyze the system performance [33,34]:

(2) $$\mathrm{Specificity}=\frac{\mathrm{TN}}{\mathrm{TN}+\mathrm{FP}}$$

(3) $$\mathrm{Precision}=\frac{\mathrm{TP}}{\mathrm{TP}+\mathrm{FP}}$$

(4) Recall=TPTP+FN

(5) $$\mathrm{Accuracy}=\frac{\mathrm{TP}+\mathrm{TN}}{\mathrm{TP}+\mathrm{TN}+\mathrm{FN}+\mathrm{FP}}$$

(6) $$\mathrm{AUC}=\frac{\mathrm{Sensitivity}+\mathrm{Precision}}{2}$$

(7) $$F=\frac{2\times \mathrm{PR}}{P+R}$$

in which TP (True Positive), TN (True Negative), FP (False Positive), and FN (False Negative) indicate, and all values are dimensionless.

Accuracy represents the proportion of samples that are correctly classified. Recall (R) is defined as the ratio of the TP samples to the sum of the TP and FN samples. Precision (P) is defined as the ratio of the TP samples to the sum of the TP and FP samples.

## 3. Results

### 3.1. Results of ANOVA for Sugar and Carbohydrate Content of Potato Cultivars

The results of analysis of variance (ANOVA) for sugar and carbohydrate contents of five different potato cultivars were significant at the level of 1%, the mean of squares values of sugar and carbohydrate were 2.198 and 8184.567, respectively, with coefficients of variation of 0.270 CV and 7.671 CV, respectively.

**Figure 3:**
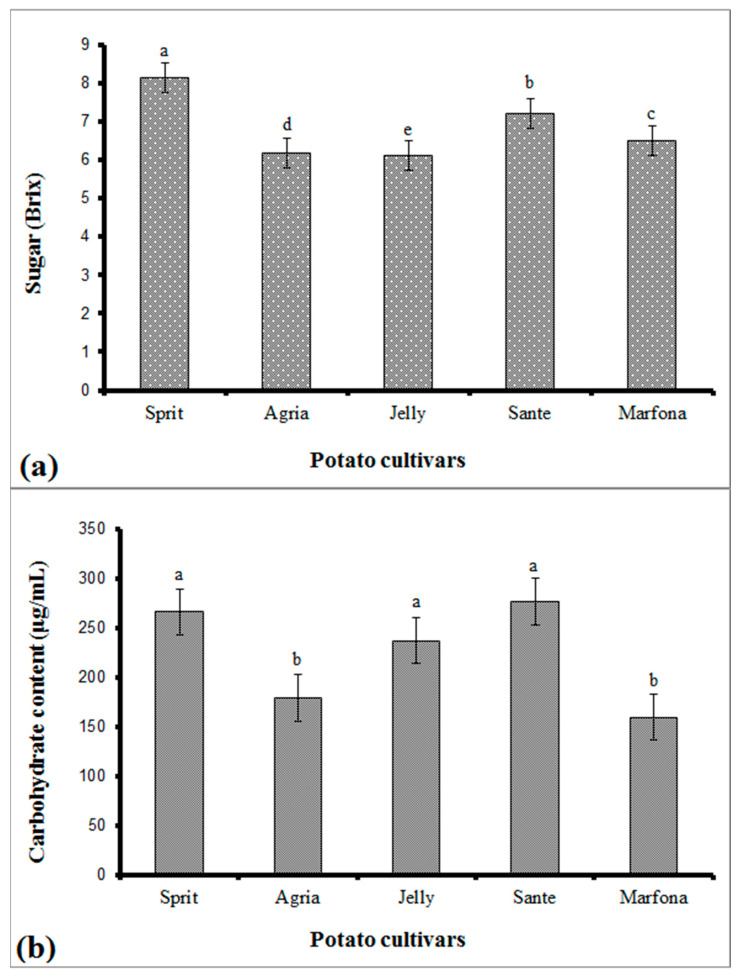
Result of Duncan mean comparison test for the (**a**) sugar content and (**b**) carbohydrate of potato cultivars (α = 0.010). The letters a, b, c, d, e describe significant differences between the obtained means. Means with the same letters do not differ significantly.

The average sugar content in potato cultivars by Brix index (grams of sugar per 100 g of solution) is shown in Figure 3a. With respect to the shape parameter, the Sprite cultivar has the highest content of sugar (8.151). However, Agria and Jelly cultivars have the lowest amount of sugar content (6.180 and 6.122 Brix, respectively).

In addition, the average potato carbohydrates’ content in potato cultivars can be seen in Figure 3b. According to the results, the highest carbohydrate content was observed in Sprite, Sante, and Jelly cultivars equal to 277, 266, and 237 μg/mL, respectively. However, the least carbohydrate content was recorded for Marfona and Agria cultivars.

### 3.2. E-Nose Result

**Figure 4:**
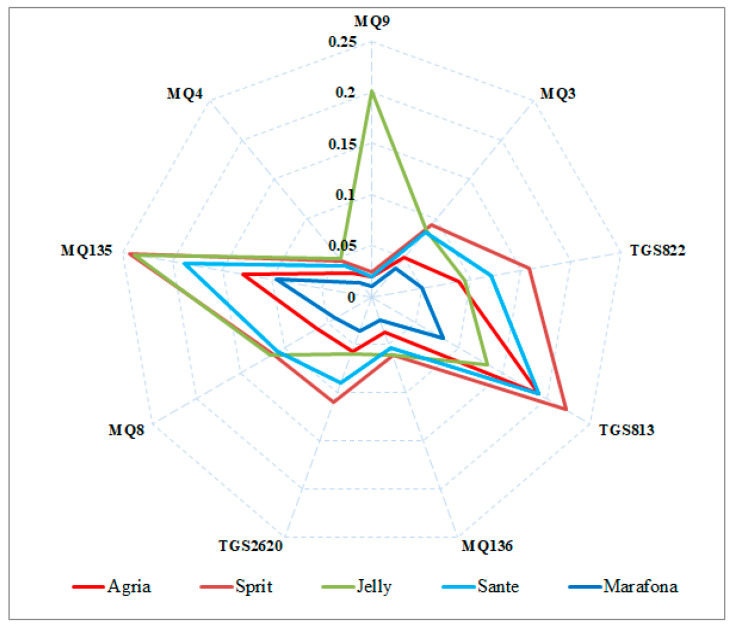
Radar raw fingerprint chart (sensor intensities) of the VOCs potato cultivars.

The experiments were performed to identify five different potato cultivars. Radar diagrams were used to observe the differences in patterns (fingerprints) between different potato cultivars. The average output data of electronic nose sensors, during 100 s of measurements, are plotted as a radar diagram following the normalization using Equation (1) (Figure 4). Using this diagram, it is possible to visualize the difference between the response patterns of the sensors to the odor of each potato cultivar. As can be seen from Figure 3, there is considerable similarity in the fingerprints of different potato cultivars. Except for the Jelly cultivar, whose pattern is somewhat different from other cultivars, all other cultivars have almost the same pattern but are varied in values. Accordingly, the highest odor is related to Jelly, Sprite, and Sante cultivars. These three cultivars also have the highest carbohydrate content. It can probably be said that the reason for the greater odor of these three cultivars is due to their higher carbohydrate content.

**Figure 5:**
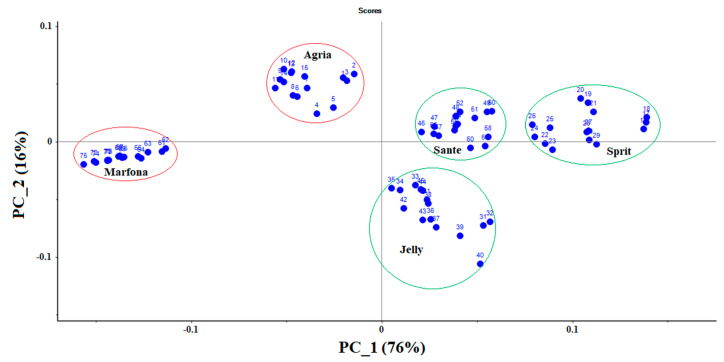
Two-dimensional PCA plot to identify five different potato cultivars with data collected using an electronic nose.

The scores diagram (Figure 5) shows the total variance of the data equal to PC-1 (76%) and PC-2 (16%), respectively, with the first two principal components, constitute 92% of the total variance of the normalized data. When the total variance is above 60%, it means that the first two PCs are sufficient to explain the total variance of the dataset. According to the figure, the three cultivars Gelly, Sprite, and Sante, with higher carbohydrate contents, can be seen on the right side of the score diagram, and the two cultivars, Marfona and Agria, can be seen on the left side of this diagram. It can be assumed that the e-Nose has a good response to the odor of carbohydrates, and it may be possible to distinguish different potato cultivars only based on their carbohydrate contents. It indicates the high accuracy of the electronic nose in detecting the odor of different products.

**Figure 6:**
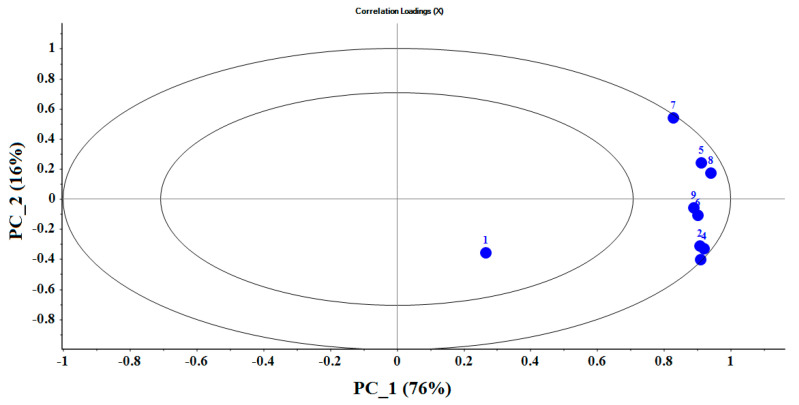
Loading plot for PCA analysis to identify five different potato cultivars. Abbreviations: (1) MQ9, (2) MQ4, (3) MQ135, (4) MQ8, (5) TGS2620, (6) MQ136, (7) TGS813, (8) TGS822, and (9) MQ3.

In the correlation loadings plot, the relations between all variables can be shown. The loading diagram (Figure 6) shows the relative contribution of the sensors for each principal component. The inner ellipse shows 50%, and the outer ellipse shows 100% of the variance of total data. The higher the loading coefficient of the sensor, the higher the contribution of that sensor in detection and classification. Therefore, it can be said that sensors on the outer circle has a greater role in data classification.

### 3.3. LDA and ANN Results

**Figure 7:**
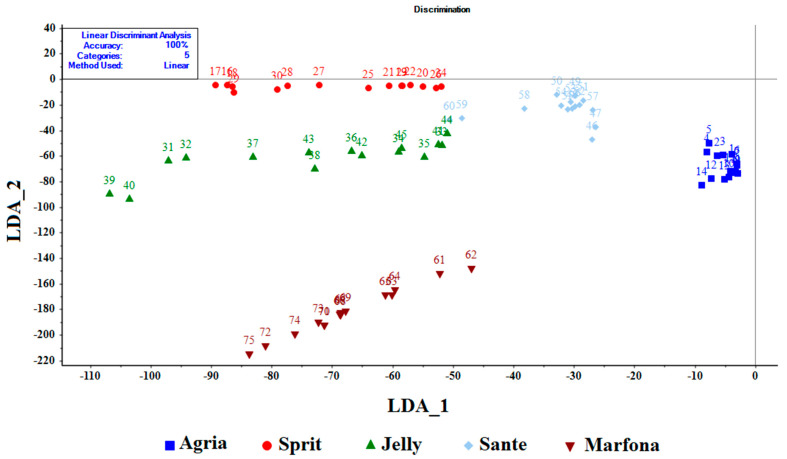
LDA analysis to identify five different potato cultivars.

LDA and ANN methods were used to recognize the potato cultivars, based on sensor output response. Unlike the PCA method, the LDA method is able to extract multiple sensor information to optimize the resolution between classes. Therefore, this method was used to detect five potato cultivars, based on the output response of the sensors. The result of the identification of cultivars was obtained 100% (Figure 7).

The lowest value of cross-entropy obtained during the training period was 2.1%. A cross-entropy error of less than 1% was proposed to serve as a stopping condition in training the e-nose. In this study, it was stated that if the %CE training was more than 1%, the system would be re-trained and more samples would be added, until the required cross-entropy error is reached. Table 3 shows the confusion matrix for the classification of five potato cultivars.

In the confusion matrix, the rows correspond to the actual classes and the columns to the identified classes. Oblique cells correspond to correct classified observations, and non-oblique cells correspond to incorrect classified observations. Table 3 shows the confusion matrix results from the recognition of potato cultivars using LDA and ANN methods. Statistical results of the artificial neural network classification models, developed using the e-nose outputs as inputs for the classification of five varieties of potatoes are shown in Table 4.

The oblique cells of the confusion matrix were composed of the correct number and percentage of classification. For example, the first cell corresponding to the Agria cultivar was correctly classified with 20% of all 75 datasets observed. Since there was no case of incorrect classification in the LDA method, the classification accuracy was 100%.

Furthermore, the value of the receiver operating characteristic (ROC) was very sensitive for the classification of the five potato cultivars (true positive rate; 0.960).

Table 5 shows the testing results for classifiers challenged with a new dataset of potato samples; statistical results were reported using Equations (2)–(7). Among the models tested, both linear and quadratic LDA classifiers provided the best performance, including recall and specificity. The classification recalls of these five categories (with the ANN and LDA method) were 96 and 100%, respectively, while their precision percentages were 96.666 and 100%, respectively. Using the precision and recall percentage values, shown in the gray cells in the last row and rightmost column of the matrix, respectively, the computed F-measure of these four categories were 95.923%, and 100%, respectively. Despite the fact that Precision and Recall are valid metrics in their own right, one can be optimized at the expense of the other; therefore, the F-Measure was used. In the case of the five-potato cultivar classification prediction, in most experimental proofs, the e-nose was able to correctly correlate the input data with the actual input concentrations. The results show that the overall accuracy of the electronic nose, when using it in the classification of five potato cultivars, was very satisfactory.

## 4. Discussion

According to the results, the highest carbohydrate content was observed in Sprite, Sante, and Jelly cultivars, equal to 277, 266, and 237 μg/mL, respectively; the Sprite cultivar had the highest content of sugar (8.151). The difference in the sugar content of different cultivars is due to the differences in the hydrolysis of starch (the main component of potato tubers), which occurs as a result of the crop respiration; the lower the starch content in a cultivar, the less sugar has the cultivar. It is important to note that the chemical composition depends on the potato cultivar, soil, climate, and agronomic factors. In general, it can be said that potatoes with more sugar content are suitable for the chips industry and potatoes with medium sugar content are suitable for frying [35].

Gumul et al. [36] measured the sugar content for five different potato cultivars. They also stated that the lower the sugar content of different potato cultivars, the lower the quality of the product, because at high temperatures, sugars react with Maillard to form potential substances that are dangerous to human health. This reaction is also observed during the thermal processing of food [23]. Rutolo et al. [37] studied the detection of potato soft rot caused by pectobacterium carotovorum using an array of low-cost gas sensors. Their goal was to investigate the potential of a set of low-cost gas sensors to diagnose the disease. In laboratory conditions, they analyzed 80 potatoes with and without soft rot by an array of 11 different gas sensors. The results showed that 100% detection accuracy can be achieved with only three sensors.

Rasekh and Karami [26,27] reported similar results for predicting fruit juice fraud. In addition, the results obtained in this study were far higher than the values reported by Ayari et al. [38] for the detection of oxidation in animal and herbal oils. These results were also more accurate than the research by Yu et al. [39] on the classification of green tea, based on data provided by electronic nose. Rusinek et al. [40] described the differences in the quality of stored rapeseed, during which there was a loss of material quality. The electronic nose device has been successfully used to determine differences in coffee grades, based on aroma studies [32,41]. In these works, advanced statistical methods were used to describe the relationships between the obtained parameters. Similar results have been reported by other researchers [42,43,44,45].

## 5. Conclusions

This paper reports on the use of MOS gas sensors analysis equipment to detect and investigate odors associated with potato cultivars. Given that there is currently no cost effective, non-destructive, reliable, and practical approach for the classification and identification of potato cultivars, this method has the potential to be used as a fast and non-destructive method to identify different potato cultivars. Using this method for identifying potato cultivars will be very useful for researchers to select and produce pure cultivars and for farmers to produce uniform and certified crops.

The large number of potato varieties, lack of awareness about its new cultivars among farmers to cultivate, time-consuming and inaccurate process of identifying different potato cultivars, and the significance of identifying potato cultivars and other agricultural products (in every food industry process) all necessitate new, fast, and accurate methods. It will be very useful for researchers to select and produce pure cultivars and for farmers to produce uniform and certified crops. Therefore, the results of this study can be effective in the rapid identification of potato cultivars.

## Figures and Tables

**Table 1:** Carbohydrate values obtained for different potato cultivars.

Variety	Absorption Wavelength (nm)			Carbohydrate Content (μg/mL)		
	1	2	3	1	2	3
Sprit	0.780	0.793	0.840	258	262	278
Agria	0.492	0.543	0.573	165	181	191
Jelly	0.675	0.714	0.761	223	236	252
Sante	0.804	0.808	0.901	265	268	297
Marfona	0.401	0.461	0.561	136	155	187

**Table 2:** The sensor types, gas detection ranges, and known chemical sensitivity of tin oxide MOS sensors within the electronic nose sensor array.

Row	Sensor Name	Detection Ranges (ppm)	Main Applications (Gas Detector)
1	MQ9	10–1000 and 100–10,000	CO and combustible gas
2	MQ4	300–100	Urban gases and methane
3	MQ135	10–10,000	Steam ammonia, benzene, sulfide
4	MQ8	100–1000	Hydrogen
5	TGS2620	50–5000	Alcohol, steam organic solvents
6	MQ136	1–200	Sulfur dioxide (SO_2_)
7	TGS813	500–10,000	CH_4_, C_3_H_8_, C_4_H_10_
8	TGS822	50–5000	Steam organic solvents
9	MQ3	10–300	Alcohol

**Table 3:** Confusion matrix to identify five different potato cultivars using LDA and ANN methods.

Model	Variety	Agria	Sprit	Jelly	Sante	Marafona	
LDA	Agria	15 20%	0 0%	0 0%	0 0%	0 0%	100% 0%
	Sprit	0 0%	15 20%	0 0%	0 00%	0 0%	100% 0%
	Jelly	0 0%	0 0%	15 20%	0 0%	0 0%	100% 0%
	Sante	0 0%	0 0%	0 0%	15 20%	0 0%	100% 0%
	Marafona	0 0%	0 0%	0 0%	0 0%	15 20%	100% 0%
		100% 0%	100% 0%	100% 0%	100% 0%	100% 0%	100% 0%
ANN	Agria	12 16%	0 0%	0 0%	0 0%	0 0%	100% 0%
	Sprit	0 0%	15 20%	0 0%	0 0%	0 0%	100% 0%
	Jelly	0 0%	0 0%	15 20%	0 0%	0 0%	100% 0%
	Sante	3 4%	0 0%	0 0%	15 20%	0 0%	83.333% 16.667%
	Marafona	0 0%	0 0%	0 0%	0 0%	15 20%	100% 0%
		80% 20%	100% 0%	100% 0%	100% 0%	100% 0%	96% 4%

**Table 4:** Statistical results of the artificial neural network classification models, developed using the e-nose outputs as inputs for the classification of five varieties of potatoes. Abbreviations: CE: means Cross entropy.

Stage	Samples	Accuracy	Error	CE
Training	45	97.801	2.202	0.455
validation	15	93.324	6.711	0.902
Testing	15	93.314	6.736	0.917
Overall	75	96.001	4.000	0.065

**Table 5:** Performance parameters of LDA and ANN models.

Models	Accuracy	Precision	Recall	Specificity	AUC	Fscore
**LDA**	1.000	1.000	1.000	1.000	1.000	1.000
**ANN**	0.984	0.966	0.960	0.990	0.978	0.959

## Data Availability

The datasets used and/or analyzed during the current study are available from the corresponding author on reasonable request.
